# Pedigree analysis of 220 almond genotypes reveals two world mainstream breeding lines based on only three different cultivars

**DOI:** 10.1038/s41438-020-00444-4

**Published:** 2021-01-01

**Authors:** Felipe Pérez de los Cobos, Pedro J. Martínez-García, Agustí Romero, Xavier Miarnau, Iban Eduardo, Werner Howad, Mourad Mnejja, Federico Dicenta, Rafel Socias i Company, Maria J. Rubio-Cabetas, Thomas M. Gradziel, Michelle Wirthensohn, Henri Duval, Doron Holland, Pere Arús, Francisco J. Vargas, Ignasi Batlle

**Affiliations:** 1 Institut de Recerca i Tecnologia Agroalimentàries (IRTA), Mas Bové, Ctra. Reus-El Morell Km 3,8, 43120 Constantí, Tarragona Spain; 2 Institut de Recerca i Tecnologia Agroalimentàries (IRTA), Centre de Recerca en Agrigenòmica (CRAG), CSIC-IRTA-UAB-UB. Cerdanyola del Vallès (Bellaterra), 08193 Barcelona, Spain; 3 Centro de Edafología y Biología Aplicada del Segura, Consejo Superior de Investigaciones Científicas (CEBAS-CSIC), P.O. Box 164, 30100 Espinardo, Murcia Spain; 4 Institut de Recerca i Tecnologia Agroalimentàries (IRTA), Fruitcentre, PCiTAL, Gardeny Park, Fruitcentre Building, 25003 Lleida, Spain; 5 Centro de Investigación y Tecnología Agroalimentaria de Aragón (CITA), Avda. Montañana 930, 50059, Zaragoza, Instituto Agroalimentario de Aragón IA2 (CITA-Universidad de Zaragoza), Zaragoza, Spain; 6 University of California, 1 Shields Avenue, Davis, CA 95616 USA; 7 University of Adelaide, Waite Research, School of Agriculture, Food and Wine, PMB 1, Glen Osmond, Adelaide, SA 5064 Australia; 8 Institut National de la Recherche Agronomique (INRA), Domain St. Maurice CS 60094, 84143 Montfavet Cedex, France; 9 Agricultural Research Organization, Newe-Ya’ar Research Center, P.O. Box 1021, Ramat Yishad, 30095 Israel

**Keywords:** Inbreeding, Plant breeding

## Abstract

Loss of genetic variability is an increasing challenge in tree breeding programs due to the repeated use of a reduced number of founder genotypes. However, in almond, little is known about the genetic variability in current breeding stocks, although several cases of inbreeding depression have been reported. To gain insights into the genetic structure in modern breeding programs worldwide, marker-verified pedigree data of 220 almond cultivars and breeding selections were analyzed. Inbreeding coefficients, pairwise relatedness, and genetic contribution were calculated for these genotypes. The results reveal two mainstream breeding lines based on three cultivars: “Tuono”, “Cristomorto”, and “Nonpareil”. Descendants from “Tuono” or “Cristomorto” number 76 (sharing 34 descendants), while “Nonpareil” has 71 descendants. The mean inbreeding coefficient of the analyzed genotypes was 0.041, with 14 genotypes presenting a high inbreeding coefficient, over 0.250. Breeding programs from France, the USA, and Spain showed inbreeding coefficients of 0.075, 0.070, and 0.037, respectively. According to their genetic contribution, modern cultivars from Israel, France, the USA, Spain, and Australia trace back to a maximum of six main founding genotypes. Among the group of 65 genotypes carrying the *S*_*f*_ allele for self-compatibility, the mean relatedness coefficient was 0.125, with “Tuono” as the main founding genotype (24.7% of total genetic contribution). The results broaden our understanding about the tendencies followed in almond breeding over the last 50 years and will have a large impact into breeding decision-making process worldwide. Increasing current genetic variability is required in almond breeding programs to assure genetic gain and continuing breeding progress.

## Introduction

Almond [*Prunus dulcis* (Miller) D.A. Webb, syn. *P. amygdalus* (L) Batsch] is the most economically important temperate tree nut crop worldwide. Due to increasing demand, production areas are expanding into warm and cold climatic regions of both hemispheres. Almond world production (1,258,324 kernel tonnes) is led by the USA (80%), Australia (6%), and Spain (5%)^1^.

The origin of almond within the Amygdalus subgenus, including cultivated almond and its wild relatives such as *P. fenzliana* Fritsh, *P. bucharica* (Korsh.) Fedtsch, *P. kuramica* (Korsh.) Kitam., and *P. triloba* Lindl^2,3^ took place ~5.88 million years ago^4^. Almond originated in the arid mountainous regions of Central Asia, where it was first cultivated around 5000 years ago^5^ and then moved to the Mediterranean region and later to California and the southern hemisphere (South America, Australia, and South Africa)^6^. Wide cultivation of almond, often under the more severe environments of Central Asia and the Mediterranean region, was possible because of the availability of a highly diverse gene pool, genetic recombination promoted by its self-incompatibility, and possibly, by interspecific hybridization and gene introgression involving other members of the Amygdalus subgenus. As a result, almond is an extremely variable species, with a high morphological and physiological diversity. This variability, measured with biochemical and molecular markers^7–9^, has revealed that almond is the most genetically variable of the diploid *Prunus* cultivated species^10,11^.

In the Mediterranean Region, 2000 years of almond culture concentrated production to specific areas, where well-defined seedling ecotypes and local cultivars evolved^2^. By the turn of the 20th century, most of these almond-producing countries had identified locally desirable cultivars that were often seedling selections of unknown origin^12^. Thus, growers selected cultivars and landraces, which represented a rich genetic diversity. Most of these Mediterranean local cultivars have largely disappeared from cultivation in the last 50 years^13^. Modern almond cultivation is based on a reduced number of cultivars (preferably self-compatible) grafted onto soil-adapted clonal rootstocks and cultivated under irrigated conditions when possible.

Modern almond breeding started in the 1920s with the making of controlled crosses and seedling selections to meet changing agronomic and market demands. Currently, there are six active public breeding programs worldwide: the USA (UCD-USDA), Spain (CITA, IRTA, and CEBAS-CSIC), Australia (University of Adelaide), and Israel (ARO). Some private breeding programs exist also in the USA. In addition, there were various breeding initiatives in Russia, France, Greece, Italy, and Argentina^13^. Different breeding objectives were developed according to regional agronomic, commercial, and market requirements. One of the main differences in the objectives is nut shell hardness. Two types of almonds are bred: soft-shelled (in the USA and Australia mainly) and hard-shelled (in most Mediterranean countries). Common aims of Mediterranean breeding programs are self-compatibility and late-blooming, as most traditional almond cultivars are self-incompatible and early-blooming. Self-compatibility is controlled by a single self-compatibility *S*_*f*_ dominant allele^14^. During the last 50 years, almond breeding for self-compatibility has mainly used two sources of *S*_*f*_, local landraces originated in Italy (“Tuono” and “Genco”) and related species such as *P. persica* and *P. webbii*^15^.

Almond breeders have relied mainly on outcrossing and, occasionally, on introgression from other *Prunus* species, for the development of new cultivars. Initially, in the USA (with limited accessible genetic resources) and later in Russia and Mediterranean region (with more diverse germplasm available), rapid genetic advances were achieved. In California, “Carmel” (introduced in 1966), as “Nonpareil” pollinizer, was the first cultivar release with extensive commercial impact. In Russia and the former Soviet Union, several late-flowering and frost-hardy cultivars were obtained in the 1950s with Primorskyi (date unknown) later used extensively for breeding in Europe. In the Mediterranean region, late flowering, productive, well-adapted, and resilient cultivars like Ferragnès (1973) or Masbovera (1992) were released with great success. The French self-compatible cultivar Lauranne (1991) showed a broad environmental adaptation, high production, and regular cropping.

Although improved cultivars continued to be released, the amount of progress per generation diminishes since parents were continually drawn from the same gene pool^13^. This situation has resulted in a potential loss of genetic variability in new breeding stocks and cultivars. Inbreeding depression in almond, expressed as low vigor, reduced flower number and fruit set, increased fruit abortion, lowered seed germination and seedling survival, increased leaf and wood abnormalities, and loss of disease resistance have been reported^16–19^. In addition, low self-fruitfulness in self-compatible almond genotypes was suspected to be due to inbreeding^20^.

Regarding breeding for self-compatibility, male parents carrying the *S*_*f*_ allele and sharing the other *S* allele with the female parent are commonly used. In addition, crossing heterozygous self-compatible parents in breeding programs has been suggested to obtain homozygous self-compatible genotypes to be used in further breeding^21^. Such breeding strategies can narrow the genetic variability of crops when they lead to a reduced number of genotypes utilized as parents.

Summarizing, modern almond breeding and production are dominated by a small number of widely distributed and related cultivars. This situation can lead to a potential increase of inbreeding depression and genetic vulnerability, i.e., susceptibility of most of the grown cultivars to biotic and abiotic stresses due to similarities in their genotypes^22,23^. Therefore, it is needed to have up-to-date information of the relationships among genotypes used at breeding and production levels.

Several almond populations have been analyzed with molecular markers in order to determine genetic variability and relatedness^9,24–26^. However, these studies were performed with material from limited geographic areas and do not represent the current worldwide status of almond breeding stocks. Although genomic measures of inbreeding are more accurate than those obtained from pedigree data^27,28^, pedigree-based analysis is a cost-effective technique to estimate these parameters in breeding populations and an alternative when genomic measures are unviable. Several reports have evaluated inbreeding based on pedigree data in breeding populations of fruit and nut tree crops^29–32^. In almond, a pedigree analysis of 123 different genotypes from the USA, France, Spain, Israel, and Russia was reported^33^. However, their work was mainly focused on North American genotypes and did not include many cultivars that have subsequently been released worldwide. This study aimed to determine the genetic structure of current breeding stocks and breeding tendencies over the last 50 years using marker-verified pedigree data.

## Materials and methods

### Marker-verified pedigree data

Pedigree data of 220 almond genotypes (169 of known origin and 51 of unknown origin) were compiled from available bibliography and breeding records. From the 220 almond genotypes, 37 genotypes were no longer available (17% of the studied genotypes) as they were eliminated some time ago or were from discontinued breeding programs. To verify parental relationships of the rest of genotypes (183), we used SSRs, SNPs, and self-incompatibility S-allele data from previous studies performed by the breeding programs taking part in this study (Supplementary Material 1). Marker data confirmed both parents of 71 genotypes and one parent of four genotypes (146 confirmed relationships) and found three erroneous parentages. Two wrong parentages were found on the male parent of “Capella” and “Davey”, changing their pedigree to open-pollinated and a third incorrect parentage on “Yosemite” female parent, eliminating this genotype from the analysis.

After the corrections made, pedigrees of 169 genotypes of known origin (77 of them marker-verified, approximately 54% of the available genotypes) were analyzed (Supplementary Material 1). The origin of the genotypes were 59 from Spain, 56 from the USA, 16 from Russia, 11 from Israel, 10 from France, 7 from Australia, 7 from Greece, 2 from Argentina, and 2 from Italy.

A pedigree data file was created. Each record in the file contained one cultivar or selection name, the female parent and the male parent, in that order. Once entered, these data were available for inbreeding analyses such as determining the number of times a cultivar appeared in a pedigree as a male or female genitor. Genotypes of known origin were classified into two groups according to self-compatibility: 104 self-incompatible and 65 self-compatible.

### Inbreeding coefficient, pairwise relatedness, and genetic contribution

The inbreeding coefficient (*F*) is defined as the probability that a pair of alleles at any locus in an individual are identical by descent, and it is calculated by the following formula^34^:

$$F_x = {\sum} {\left[ {\left( {\frac{1}{2}} \right)^{n_1 + n_2 + 1}\left( {1 + F_A} \right)} \right]},$$

where *n*_1_ = number of generations from one parent back to the common ancestor, *n*_2_ = number of generations from the other parent back to the common ancestor, and *F*_*A*_ = inbreeding coefficient of the common ancestor.

Pairwise relatedness (*r*) or coancestry coefficient, the degree of relationship by descent of two parents, equals the inbreeding coefficient of their prospective progeny.

The genetic contribution (*GC*) of a founder to a cultivar is calculated by the following formula^35^:

$$GC = \mathop {\sum}\limits_1^x {\left( {\frac{1}{2}} \right)^n},$$

where *n* = number of generations in a pedigree pathway between the founding clone and the cultivar and *x* = number of pathways between the founding clone and the cultivar. The three parameters were calculated using the SAS INBRED procedure (SAS 9.4 SAS Institute, Cary, NC, USA).

In summary, the inbreeding coefficient measures the probability that two alleles in a locus are identical by descent and so copies of the same allele from a previous generation. The pairwise relatedness measures the probability that two alleles at any locus are identical by descent (copies of the same allele in a previous generation) between two different individuals. *F* and *r* range from 0 to 1, with values close to 0 indicating a low degree of inbreeding or relatedness and values close to 1 indicating a high degree of inbreeding or relatedness. The genetic contribution estimates the proportion of genome that comes from the same individual. Thus, a child will have 0.5 genome of either parent and a grandchild will have 0.25 genomes of his grandparents.

### Analysis description

To calculate *F*, *r*, and *GC*, parents of unknown origin were assumed to be unrelated and noninbred. The seed parent involved in all open pollinations was also assumed to be unrelated to the pollen parent. These assumptions, based on the fact that most almond cultivars are obligate outcrossers because of their self-incompatibility, may lead to an underestimation of inbreeding. In the cases of genotypes of open-pollinated origin (OP), numbers OP1, OP2, and OP3 were given to the pollen parent in order to be distinguishable for genetic studies. Also, all mutants were considered to have no genetic differences from the original cultivar, thus *GC* = 1. Since the differences between such mutants and the original cultivar are expected to be caused by a few mutations in the DNA, this simplification avoids the overestimation of inbreeding coefficients. Cultivars like Supernova and Guara were considered as “Tuono” clones^36,37^. Regarding the different clones of the French paper-shell cultivar Princesse, used in both the USA and Russian breeding programs, we adopted the approach of Lansari et al.^33^ by analyzing both clones as the same cultivar. Historical reports suggest that the Hatch series “Nonpareil”, “I.X.L.”, and “Ne Plus Ultra” were seedling selections from an open-pollination progeny of the early-introduced cultivar Princesse. This cultivar probably originated from the Languedoc region in France^6,38–40^. Also, “Nikitskij” was selected in France in 1902^41^. Because their specific origins remain uncertain, we analyzed these genotypes as nonrelated, which, however, could lead to an underestimation of inbreeding.

Pedigree data were analyzed at four levels: worldwide, by country (Australia, France, Israel, Spain, and the USA), by breeding program (when different programs exist within a country: CITA, IRTA, CEBAS-CSIC, and UCD-USDA), and by genotypes carrying the *S*_*f*_ allele for self-compatibility.

## Results

### Founding clones

**Fig. 1:**
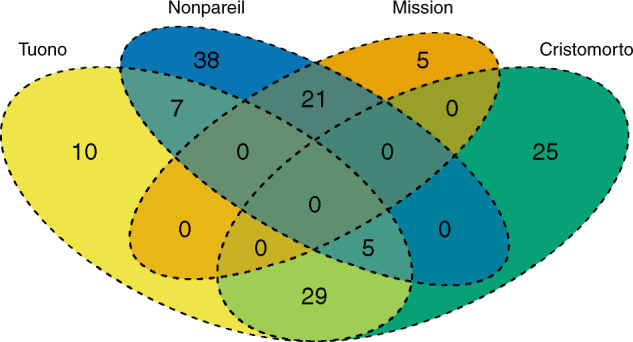
Descendants shared by “Tuono”, “Nonpareil”, “Mission” and “Cristomorto”. Venn diagram showing the number of descendants shared by “Tuono”, “Nonpareil”, “Mission” and “Cristomorto”

The entire almond pedigree traced back to 51 founding clones (Supplementary Fig. 1). “Nonpareil”, “Cristomorto”, “Mission”, and “Tuono” were the founders with the largest number of descendants in the pedigree: 140 of the 169 genotypes of known parentage traced back to one or more of these founding clones (Fig. 1). No genotype was derived from all four cultivars, i.e., did not trace back to the four founding clones. There were only five genotypes that came from a three-way shared progeny, all of them tracing back to “Tuono”–”Cristomorto“–”Nonpareil”. The largest two-way shared genotype sub in set were “Tuono”–”Cristomorto” and “Nonpareil”–”Mission” with 29 and 21 descendants, respectively. “Mission” only shared progeny with “Nonpareil” (Fig. 1).

Analyzing the results by country, breeding programs from the USA had two main founding clones, “Nonpareil” and “Mission”, with 46 and 24 descendants, respectively, out of 56. These two founders were followed by “Eureka” and “Harriott”, with 14 and 11 descendants each. Breeding programs from Spain had three main founding clones, “Tuono”, “Cristomorto”, and “Primorskyi”, with 32, 31, and 24 descendants, respectively. Cultivars from the discontinued French program had three main founding clones from two geographical origins, “Cristomorto” and “Tuono” (from Italy) with nine and five descendants, respectively, and “Aï” (from France), with eight descendants. The Australian program had only two main founding clones, “Nonpareil” and “Lauranne”, with six and five derived genotypes, respectively. The Israeli breeding program showed the most balanced pedigree with six main founding clones, “Marcona”, “Greek”, “Um ElFahem”, “Tuono”, “Nonpareil”, and “Ferragnès”.

The UCD breeding program had “Nonpareil” as the main founding clone with 29 descendants. Cultivars Eureka, Mission, and Harriott had a slight influence on the pedigree with 14, 12, and 10 descendants, respectively. Within Spain, CITA breeding program had Italian “Tuono” as the main founding clone with seven descendants. The IRTA breeding program showed three main founding clones, “Cristomorto”, “Primorskyi”, and “Tuono” with 30, 19, and 16 descendants, respectively. The CEBAS-CSIC breeding program had three main founding clones, “Tuono”, “Ferragnès”, and “Primorskyi” with 15, nine, and eight descendants, respectively. The French local cultivar Aï was also present in the three Spanish programs through the largely used French “Ferraduel” and “Ferragnès”. These two cultivars were the ancestors of 25 genotypes.

Analyzing the 65 genotypes carrying the *S*_*f*_ allele for self-compatibility, the founding clones that traced back to the origin of this allele are “Tuono”, “Genco”, and genotypes originated from introgression crosses with *P. persica* and *P. webbii*.

### Inbreeding coefficients

The mean inbreeding coefficient (*F*) of the 169 genotypes of known parentage analyzed was 0.041 (Supplementary Material 2). Some 43 genotypes presented an *F* > 0, with 14 over 0.250 (Table 1).

**Table 1:** Genotypes with the highest inbreeding coefficient

Line nanme	Female parent	Male parent	Origin	Country	Inbreeding
A2-198	C1328	C1328	CEBAS-CSIC	SPAIN	0.5
Solano	21–19 W	22–20	UCD	USA	0.375
Sonora	21–19 W	22–20	UCD	USA	0.375
Vesta	Nonpareil	Solano	UCD	USA	0.375
Ferralise	Ferraduel	Ferragnès	INRA	FRANCE	0.25
FGFD2	Ferragnès	Ferraduel	INRA	FRANCE	0.25
21–19W	Nonpareil	A1-30	UCD	USA	0.25
22–20	Nonpareil	A1-30	UCD	USA	0.25
6–27	Nonpareil	Jordanolo	UCD	USA	0.25
Calif. 24–6	Eureka	A5-25	UCD	USA	0.25
Emerald	Mission	S2	PRIVATE	USA	0.25
Profuse	Nonpareil	Jordanolo	PRIVATE	USA	0.25
Supareil	Nonpareil	Carmel	PRIVATE	USA	0.25
D01-462	A2-198	S5133	CEBAS-CSIC	SPAIN	0.25

Considering the results within each country, programs showing more inbreeding were France, the USA, and Spain with 0.075, 0.070, and 0.037 mean *F*, respectively (Supplementary Material 2). The programs from Australia and Israel had *F* = 0. The USA accessions ranged from *F* = 0 to 0.375 with 21 of the 56 genotypes having *F* > 0. The French cultivar Ferralise and selection FGFD2, derived from the same reciprocal cross, had *F* = 0.250. The Spanish selection A2-198 from CEBAS-CSIC, showed the highest inbreeding coefficient (*F* = 0.500) as it is a selfing from selection C1328 and was raised to obtain homozygous *S*_*f*_*S*_*f*_ individuals.

The UCD-USDA breeding program had a mean *F* of 0.096. Within Spain, the CITA program had *F* = 0. The CEBAS-CSIC program had only three genotypes with *F* > 0, but presented an average *F* of 0.048. The IRTA program holds 15 genotypes with *F* > 0 and a mean *F* of 0.043 (Supplementary Material 2). Considering only the 65 self-compatible genotypes, they had a mean *F* of 0.042, ranging from 0 to 0.500 (Supplementary Material 2).

### Genetic contribution

**Fig. 2:**
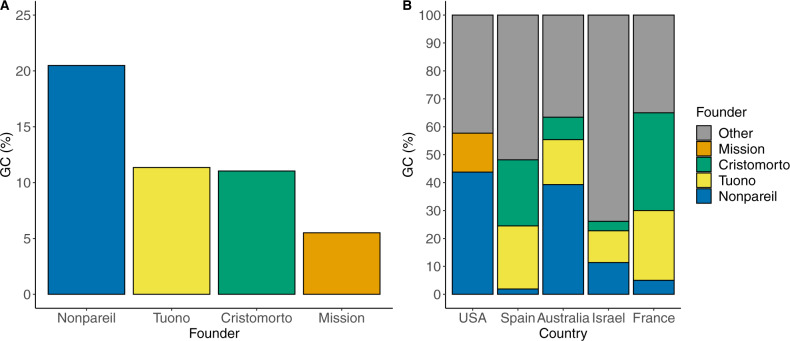
Mean genetic contribution (GC) of founders “Nonpareil”, “Tuono”, “Cristomorto”, and “Mission”. **A** Mean GC worldwide. **B** Mean GC by country

“Nonpareil”, “Tuono”, “Cristomorto”, and “Mission” were the founding clones with the highest mean genetic contribution (*GC*, Fig. 2). These four cultivars accounted for 48.4% of the total *GC* worldwide. “Nonpareil” represented 20.5% of *GC* worldwide, “Tuono” and “Cristomorto” were around 11%, and “Mission” slightly exceeded 5%. Nevertheless, the mean *GC* of these founding clones within each country was variable. The breeding programs most dependent on these founders were Australia and France, where “Nonpareil”, “Tuono”, and “Cristomorto” represented >60% of the total *GC*. Israel was the least dependent country as these founders represented ~25% of the total *GC*. Cultivar Nonpareil was the founder with the highest mean *GC* in the USA and Australia, while in Spain and France were “Tuono” and “Cristomorto”. The cultivar Mission was used only in the American programs.

Table 2 shows the *GC* of the mean founders by country. In the Australian breeding program, only two founders, “Nonpareil” and “Lauranne”, represented 71.4% of the total *GC*. The French breeding program was characterized by the extensive use of three founders “Cristomorto” (*GC* = 35.0%), “Aï” (*GC* = 30.0%), and “Tuono” (*GC* = 25.0%). These cultivars together with “Ardèchoise” and “Tardy Nonpareil” (both *GC* = 5.0%) accounted for 100% of the total *GC*. The Israeli breeding program presented six main founders, “Greek” (*GC* = 20.5%), “Marcona” (*GC* = 18.2%), “Um ElFahem” (*GC* = 13.6%), “Tuono” (*GC* = 11.4%), “Nonpareil” (*GC* = 11.4%), and “Ferragnès” (*GC* = 6.8%), which together accounted for 81.9% of the total *GC*. The USA breeding programs were largely dependent on “Nonpareil” (*GC* = 43.7%) followed by “Mission” (*GC* = 13.9%), “Eureka” (*GC* = 8.7%), and “Harriott” (*GC* = 5.5%), which all accounted for 71.8% of the total *GC*. The cultivars released by the three Spanish breeding programs were based mainly on four founders: “Cristomorto” (*GC* = 23.7%), “Tuono” (*GC* = 22.6%), “Primorskyi” (*GC* = 15.6%), and “Aï” (*GC* = 7.5%), accounting for 69.4% of the total *GC*.

**Table 2:** Genetic contribution (GC) of mean founding clones by country

Founding clone	Country of origin	GC (%)	GC total (%)
*Australia*			
Nonpareil	USA	39.3	**71.4**
Lauranne	France	32.1	
*France*			
Cristomorto	Italy	35.0	**100.0**
Aï	France	30.0	
Tuono	Italy	25.0	
Ardechoise	France	5.0	
Tardy Nonpareil	USA	5.0	
*Israel*			
Greek	Israel	20.5	**81.9**
Marcona	Spain	18.2	
Um ElFahem	Israel	13.6	
Tuono	Italy	11.4	
Nonpareil	USA	11.4	
Ferragnès	France	6.8	
*Spain*			
Cristomorto	Italy	23.7	**69.4**
Tuono	Italy	22.6	
Primorksyi	Russia	15.6	
Aï	France	7.5	
*USA*			
Nonpareil	USA	43.7	**71.8**
Mission	USA	13.9	
Eureka	USA	8.7	
Harriott	USA	5.5	

The UCD-USDA breeding program had the same founders as the overall American programs, “Nonpareil” (*GC* = 43.2%), “Eureka” (*GC* = 14.8%), “Harriott” (*GC* = 8.5%), and “Mission” (*GC* = 5.5%). Differences were observed in the use of founding cultivars between Spanish breeding programs. The CITA program was mainly based on four cultivars “Tuono” (*GC* = 35.0%), “Belle d’Aurons”, “Bertina”, and “Genco” (*GC* = 10.0% each). These cultivars were accounting for 65.0% of the total *GC*. The CEBAS-CSIC program was based also on four founders, “Tuono” (*GC* = 28.9%), “Ferragnès” (*GC* = 18.4%), “Genco” (*GC* = 12.5%), and “Primorskyi” (*GC* = 11.8%). The IRTA program was based on four founding clones too: “Cristomorto” (*GC* = 39.9%), “Primorskyi” (*GC* = 21.5%), “Tuono” (*GC* = 14.4%), and “Aï' (*GC* = 8.0%). The self-compatible Italian cultivar Tuono was the *S*_*f*_ donor most commonly used by the three Spanish programs. Within the 65 genotypes bred carrying the *S*_*f*_ allele, the 24.7% of the total *GC* came from “Tuono” (Supplementary Material 3).

### Pairwise relatedness

Pairwise relatedness (*r*) between all cultivars and breeding selections is shown in Supplementary Material 4. Cultivars with the highest mean *r* worldwide are present in Table 3. The genotype with the highest mean *r* was “Nonpareil” followed by its mutants (“Tardy Nonpareil”, “Jeffries”, and “Kern Royal”). “Vesta”, from the cross “Nonpareil” × “Solano”, was next. Carina, Mira, and Maxima (Australian genotypes originated from the cross “Nonpareil” × “Lauranne”), followed. These three genotypes were first generation of “Nonpareil”, second generation of “Tuono”, and third generation of “Cristomorto”.

**Table 3:** Genotypes with the highest mean relatedness (*r*)

Genotype	Mean *r*
Nonpareil	0.153
Tardy Nonpareil	0.153
Jeffries	0.153
Kern Royal	0.153
Vesta	0.143
A97001-1bT4	0.137
Carina	0.136
Mira	0.133
Maxima	0.133

Table 4 shows the mean *r* among breeding programs by country. Programs from Australia and France had the highest mean *r* (0.256 and 0.357, respectively). In contrast, Israel showed the lowest mean *r*. Comparing relatedness results between countries, Spain and the USA breeding programs were the least related. The most related breeding programs were those of France and Spain and also, Australia and France.

**Table 4:** Mean of pairwise relatedness (*r*) among breeding programs from five different countries

	Australia	France	Israel	Spain	USA
Australia	**0.256**	0.156	0.081	0.094	0.172
France	–	**0.357**	0.070	0.195	0.022
Israel	–	–	**0.134**	0.047	0.050
Spain	–	–	–	**0.162**	0.009
USA	–	–	–	–	**0.232**

In the Australian breeding program, the selection A97001-1BT47 had the highest mean *r* with a value of 0.375. “Rhea” was not related with the rest of the genotypes, so its mean *r* was zero. The rest of the genotypes have a mean *r* between 0.188 and 0.333 showing a high degree of relationship.

In the French breeding program, “Ferralise” had the highest mean *r* (0.500). “Ferrastar” and “R1000” had the lowest mean *r*, 0.167 and 0.111, respectively. The rest of French genotypes had a mean *r* over 0.300, being the breeding program with the most related genotypes.

**Fig. 3:**
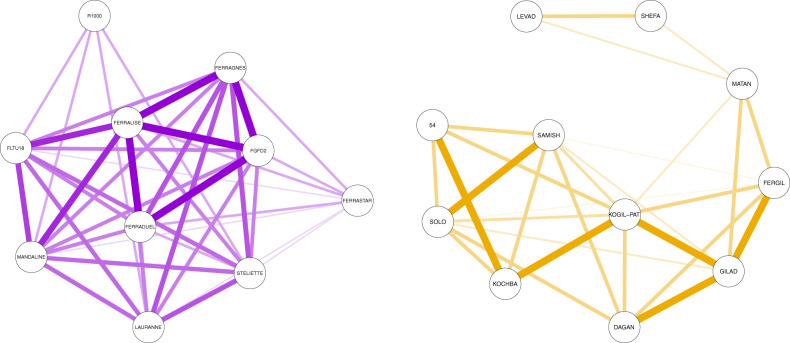
Relationship matrix of genotypes from France and Israel. Genotypes from France are showed in the left side, genotypes from Israel are showed in the right side. Line thickness shows the degree of relationship, being the thicker lines the more related genotypes

Genotypes from the Israeli program had a mean *r* under 0.225. The highest *r* observed between the ten cultivars released was 0.500 between two pairs: “Dagan”–”Gilad” and “Fergil”–”Gilad”. Selection 54 showed *r* of 0.500 with “Kochba” and 0.250 with “Kogil-Pat”, “Samish”, and “Solo”. Figure 3 compares the breeding program with the most related genotypes (France) with the breeding program with the least related genotypes (Israel).

Within the Spanish breeding programs, the highest *r* among released cultivars was 0.500 (“Antoñeta”–”Marta” and “Makako”–”Penta”). “Makako”–”Tardona” and “Penta”–”Tardona” had an *r* = 0.313. The CEBAS-CSIC’s selections A2-192 and C1328 had the highest *r* with a value of 1. In the CEBAS-CSIC program, “D01-462” had the highest mean *r* (0.273). The genotypes with a higher mean *r* in the CITA breeding program were “Guara” and “Felisia” with values of 0.278 and 0.250, respectively. The remaining CITA genotypes had a mean *r* under 0.200. Within the IRTA breeding program, the highest *r* among released cultivars was 0.563 (“Glorieta”–”Marinada”). Among IRTA’s selections, “29–47” and “35–164” showed the highest relationship with an *r* of 0.719. The selection “29–47” had the highest mean *r* (0.350). The rest of IRTA’s genotypes had mean *r* over 0.130 (Supplementary Material 4). In the USA breeding programs, “Nonpareil” and its mutations (“Tardy Nonpareil”, “Jeffries”, and “Kern Royal”) and “Vesta” had a mean *r* over 0.400. “Independence” and “Bell” had a mean *r* equal to 0. The rest of North American genotypes showed a high degree of relatedness between them. Two combinations, “Solano”–“Vesta” and “Sonora”–”Vesta”, had *r* = 1, with “Sonora”–”Vesta” *r* = 0.875. Analyzing the highest *r* values among selections and cultivars, four combinations had an *r* = 1 (“21–19 W”–“Solano”, “22–20”–“Solano”, “21–19 W”–”Sonora”, and “22–30”–“Sonora”). In addition, two other pairs: “21–19 W”–“Vesta” and “22–20”–“Vesta” had an *r* of 0.875 (Supplementary Material 4). Within the UCD breeding program, “Vesta“, “Sonora”, and “Solano” had a mean *r* over 0.400.

**Fig. 4:**
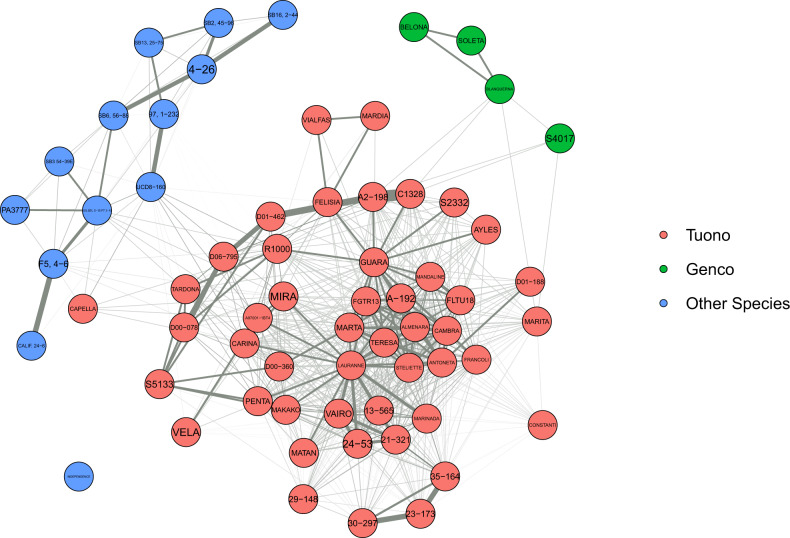
Relationship matrix of the 65 self-compatible genotypes carrying the *S*_*f*_ allele. Colors indicate origin of the *S*_*f*_ allele. Line thickness shows the degree of relationship, being the thicker lines the more related genotypes

Among the group of 65 genotypes carrying the *S*_*f*_ allele, the mean *r* was 0.125. Grouping the genotypes by origin of the *S*_*f*_ allele source (“Tuono”, “Genco”, and other *Prunus spp*), the mean *r* values were 0.210, 0.333, and 0.173, respectively (Supplementary Material 4). Figure 4 shows the main self-compatibility sources used when breeding for this character with “Tuono”, “Genco”, and other *Prunus* species involved in 48, 4, and 13 genotypes, respectively.

## Discussion

### Two mainstream breeding lines based on three different cultivars

Our genetic study of almond breeding programs worldwide demonstrated that the most widely used cultivars were Nonpareil, Tuono, Cristomorto, and Mission. “Nonpareil” had a large influence in USA and Australian programs, where soft-shelled nuts are bred. This reference cultivar was present in all the breeding programs studied (in some cases through its late-blooming mutant Tardy Nonpareil). The self-compatible “Tuono” and the late-blooming “Cristomorto” were extensively used in the Mediterranean programs, where hard-shelled nuts are bred. “Mission” initially showed a considerable importance worldwide, but deeper analysis demonstrated that it was mainly influential in private American programs. Taking into account these results, we can establish two main breeding lines based on the use of three different founders: the European programs based mainly on “Tuono” and “Cristomorto” (hard shell), and the North American–Australian programs based on “Nonpareil” (soft shell). The French and Spanish breeding programs were based directly on “Tuono” and “Cristomorto”. In the French INRA program, the Italian cultivars Tuono and Cristomorto account for 60.0% of total *GC* and were present in the pedigree of all ten cultivars and selections evaluated. Also, the local French late-flowering and *Monilinia*-resistant cultivar Aï was a parent to both “Ferragnès” and “Ferraduel”. In the three Spanish breeding programs, the importance of “Tuono” and “Cristomorto” cultivars was very high, accounting to 46.2% of total *GC*. These two cultivars were present in the pedigree of 53 out of 59 cultivars and breeding selections from Spain. These results can be explained by the large influence of the French germplasm on the Spanish breeding programs, causing a high relationship between the programs of both countries (mean *r* = 0.195). In the North American breeding programs, “Nonpareil” accounts for 43.7% of the total *GC* and was present in the pedigree of 48 out of 56 cultivars and breeding selections from the USA. In Australia, ‘Nonpareil’ accounts for 39.3% of the total *GC* and is present in the pedigree of 6 out of 7 cultivars and breeding selections. Also, “Lauranne” (32.1% of the total *GC*) reaches an importance similar to ‘Nonpareil’, explaining the close relationship between the Australian and French programs (mean *r* = 0.156). Even in other countries with noncontinuous breeding initiatives, such as Russia, Greece, or Argentina, the use of “Nonpareil” as a founder was common. Israel was the only country where these cultivars had a relatively low influence. This may be due to the extreme Israeli climatic conditions, forcing breeders to use locally adapted selections as parents. In Spain, the use of locally adapted cultivars such as Bertina at CITA as a donor for *Polystigma ochraceum (Wahlenb.) Sacc*. resistance was successful but used only to a limited extent. Other examples of secondary founders include “Primorskyi”, used regularly as late-blooming and *Fusicoccum*-resistance donor in two of the Spanish breeding programs (IRTA and CEBAS-CSIC) and “Eureka” and “Harriott” in the North American breeding programs.

### Loss of genetic variability and increasing of inbreeding at breeding and production level

Comparing our results on almond inbreeding with other *Prunus* species, the mean inbreeding coefficient worldwide of all genotypes (*F* = 0.036) was lower than that of Japanese plum^42^ and apple^43^ and several orders of magnitude lower than those calculated for peach^44,45^ and cherry^31^. Within almond, inbreeding and relatedness coefficients obtained in this study were higher than those reported by Lansari et al.^33^. While they documented only ten genotypes with *F* > 0 (four of them with *F* ≥ 0.250), we found 43 genotypes meeting this condition (14 of them with *F* ≥ 0.250). Analyzing mean *r* by country, in the case of France and the USA (with a number of cultivars comparable in both studies), this coefficient increased. This loss of variability and an associated increase of inbreeding is due to the repeated use of a limited number of parents (“Nonpareil“, “Tuono”, and “Cristomorto”) and their related genotypes, as we have shown for almond breeding.

Among the group of the 65 genotypes carrying the *S*_*f*_ allele for self-compatibility, the mean *r* was 0.125. In cherry self-compatible selections, coefficients of coancestry ranged from 0.102 to 0.256^31^ and thus were of similar magnitude. In Western Europe, the Italian cultivar Tuono was used extensively as a source of self-compatibility, late blooming, and spur-type cropping. More recently, it has become important in Israel and Australia (in Australia through “Lauranne” (“Ferragnès” × “Tuono”)). This “Ferragnès” × “Tuono” cross also originated the cultivar Steliette and was later successfully used in two of the Spanish breeding programs, resulting in three self-compatible cultivars: Cambra at CITA, and Antoñeta and Marta at CEBAS-CSIC. Thus, these five cultivars are full siblings. In addition, in the USA, breeders are using “Guara” (syn “Tuono”) as *S*_*f*_ donor. A similar case occurred in sweet cherry with the cultivar Stella as it was the most frequently utilized parent for self-compatible selections in North America^31^.

A lack of diverse germplasm may limit continued progress in almond breeding programs. This genetic limitation is of particular concern in the main producing countries. Thus, Californian and Australian production rely mainly on ‘Nonpareil’ and closely related cultivars^46,47^, while in Spain, some new Spanish cultivars like Vairo and Penta, derived from second generation of “Tuono” and “Cristomorto”, as well as “Belona” and “Soleta”, derived from second generation of “Genco”, are replacing traditional cultivars in new orchards. This trend is also favored by the almond industry needs. Only in some regions of Central Asia, Middle East, and North Africa, local and well-adapted traditional selections still play an important role in commercial production^26,48–50^.

### Usefulness of pedigree data analyzing breeding tendencies

Pedigree analysis is a cost-effective and well-established way to monitoring inbreeding and relatedness among controlled breeding populations. However, the veracity of any analysis based on this kind of data relies on the accuracy of records collected across multiple institutions and by many breeders. In order to verify parental relationships of the genotypes under study, we used SSRs, SNPs, and self-incompatibility S-allele data from previous analysis carried out by the breeding programs taking part in this study. Our molecular marker analysis confirmed 146 parentage relationships and found three errors (2% error rate), which were corrected accordingly. Thus, the marker-based pedigree analysis performed showed only small parental changes and corroborates the consistency of the results reached by this study.

However, several reports have demonstrated that large-scale genomic analysis may provide more accurate results than pedigree analysis^27,28^. This kind of genome-based pedigree analysis has already been performed in apple^51^. The recent publication of two almond reference genomes^4,52^ and the increasing availability of quality genomic data opens opportunities to complement our study and obtain more complete and accurate pedigrees based on genomic variability. This kind of studies can be useful even when some genotypes were discarded due to breeding process, as is the case in our almond pedigree work.

Although almond showed a higher genetic variability than other *Prunus* species, the historical expansion of almond from the Mediterranean region to California and from California to Australia could have caused a bottleneck effect in the breeding population under study. Different studies have reported a high genetic relatedness between Australian and Californian cultivars^9,53^, possibly caused by the introduction of a limited number of cultivars from Europe to these countries. In addition, breeding programs worldwide have used cultivars from French origin as main founders as Aï, Princesse, Ardechoise, Nonpareil, IXL, Ne Plus Ultra, or Nikitskij. This situation could have led to an underestimation of relatedness and inbreeding. The use of large-scale genomic data would provide most valuable information in this respect, expanding the almond pedigree beyond breeding records.

## Conclusions

This almond pedigree study reviews the progress made in breeding over the last 50 years. The results showed that two main breeding lineages, based on only three cultivars (Nonpareil, Tuono, and Cristomorto) have dominated modern breeding worldwide. This limitation has led to the high level of inbreeding found in modern cultivars. The inbreeding observed in our study could explain the phenotypic depression early reported in breeding populations^16–20^. Thus, future almond breeding should avoid inbreeding and favor genetic gain. Diversify the sources of self-compatibility, which are presently dominated by “Tuono”, and broaden the germplasm used when breeding is an urgent need. Additional analyses based on genomic data are needed to more accurately determine the levels of inbreeding and the loss of genetic variability among almond breeding programs worldwide.

## Supplementary information

Supplementary Figure 1

Supplementary Material 1

Supplementary Material 2

Supplementary Material 3

Supplementary Material 4
